# Psychometric evaluation of the Korean version of the sexual communication self-efficacy scale among South Korean college students

**DOI:** 10.3389/fpubh.2025.1488982

**Published:** 2025-03-31

**Authors:** Sujung Lee, Jungmin Lee

**Affiliations:** School of Nursing, Hallym University, Chuncheon, Republic of Korea

**Keywords:** adolescent, factor analysis, sexual intercourse, students, validation study

## Abstract

**Introduction:**

In this study, the validity and reliability of the cross-culturally adapted Korean version of the Sexual Communication Self-Efficacy Scale (KR-SCSES) were investigated.

**Methods:**

The participants were 227 college students enrolled for an academic year who had experienced sexual intercourse.

**Results:**

The confirmatory factor analysis showed an adequate model fit index (χ^2^ = 283.444, *df* = 132, *p* < 0.001, RMSEA = 0.07, CMIN/DF = 2.15, RMR = 0.04, TLI = 0.92, and CFI = 0.93). Furthermore, as a result of reliability verification, the Cronbach's α was 0.91, confirming that the reliability of the Korean version of the tool was very high.

**Conclusion:**

This study showed that the KR-SCSES is a valid and reliable tool for evaluating the sexual communication skills and self-efficacy of Korean university students. These results can be used to develop future interventions and awareness education programs related to risky sexual behavior among college students.

## 1 Introduction

College students' sexual problems constantly emerge as a significant societal problem that needs to be solved. Most students have their first sexual experience and actively start engaging in sexual activities during college. Kim (1) conducted a study on college students and reported that 70% had had sexual experience(s); 6 out of 10 participants had sexual intercourse for the first time before the age of 21. Moreover, they acted based on their sexual excitement and mood at the moment, and their sexual impulses were stronger than those of other age groups. At that age, the value system related to sexual behavior was not fully established. When making subjective judgments or choices about sexual behavior, individuals may be more vulnerable to problems related to sexual behavior, which may be strongly influenced by their sexual partners (2, 3).

According to previous studies, sexual partners are a significant factor influencing the intention of condom use among college students (4, 5), highlighting the social context of condom use. Based on these findings, the use of condoms by young people can be influenced by the number of sexual partners and their relationship with them, which suggests that young people are more likely to be involved in risky sexual behavior and relationships (2, 6, 7). More than half of the college students entering adulthood do not use condoms during intercourse, increasing not only their exposure to risky sexual behavior but also their risk of a negative health outcome (1). Increased premarital sex and lower contraceptive rates among college students can lead to unsafe and unprotected sex, increasing the risk of unwanted pregnancy and sexually transmitted infections (8, 9).

Using condoms during sexual intercourse requires at least some awareness and willingness on the part of both partners (10). Therefore, their use critically depends on the agreement and effective communication between partners. Sexual communication is a crucial factor influencing sexual health behaviors (4, 5, 10). Poor communication between partners may essentially contribute to risk-taking in young adults. According to previous studies, those who communicate more frequently with their partners about HIV, pregnancy, condom use, and related issues are more likely to continue contraceptive use (4, 10). Furthermore, some studies have interpreted that more frequent discussions about condom use predicted consistent condom use, whereas less frequent discussions about condoms predicted inconsistent use (11–13).

Effective sexual communication with one's partner and high self-efficacy can be essential determinants that positively affect decision-making regarding and the practice of condom use (14, 15). Studies have shown that self-efficacy and assertive sexual communication are associated with positive condom attitudes and use (16–18). Sexual assertiveness is an individual's ability to fulfill sexual desires and facilitate sexual behavior with a partner (19). Thus, efforts should be made to enhance communication quality, including sexual assertiveness and self-efficiency, and promote condom use among young individuals; this will ultimately help them protect themselves by making decisions about their sexual behavior with a sense of responsibility in situations of sexual conflict.

Overall, further studies are needed to explore how the quality of communication, self-efficacy, and sexual assertiveness influence safe sexual behavior, which could eventually affect sexual health and wellbeing among college students entering adulthood. However, the tools available to measure individuals' sexual communication self-efficacy are limited. Therefore, this study aimed to develop and evaluate the Korean version of the Sexual Communication Self-Efficacy Scale (KR-SCSES) to measure confidence in engaging in sexual activities with a sexual partner. Developing a scale and testing its psychometric properties could provide institutions and healthcare professionals with a reliable, valid, and efficient assessment of risky sexual behaviors in young adults. This study is significant because it contributes to identifying the ability of college students to attempt their desired sexual behavior, rejects unwanted sexual behavior, become pregnant, and prevent disease by incorporating new knowledge in the area of risky behaviors among young individuals.

## 2 Materials and methods

### 2.1 Design

This methodological study was conducted to explore the psychometric properties of the KR-SCSES. The H University Institutional Review Board of South Korea approved this study (HIRB-2022-010).

### 2.2 Sample

The target population of college students was recruited from April 10–16, 2023, for this study. The inclusion criteria were as follows: (1) enrolled in a college academic year, (2) having experienced sexual intercourse, and (3) being able to read and communicate in Korean.

The sample size was calculated based on DeVellis's recommendation (20). More than 200 samples were needed to obtain reliable factors for an exploratory factor analysis, which represented 10 multiples of the scale's 20 questions. Considering a dropout rate of 20%, we recruited 240 participants, and the final sample included 227 participants. The participants were recruited via announcements on Korean university online community platforms (e.g., blogs) to effectively reach the target population. They were provided with written informed consent documents that thoroughly outlined the study's purpose, procedures, risks, and benefits. Before commencing the online survey, the participants were required to indicate their agreement by clicking “yes” to provide their explicit consent. The written informed consent process was approved by the ethics committee to uphold participant rights and ensure their protection.

### 2.3 Measures

#### 2.3.1 Sexual communication self-efficacy scale

This scale can be used to assess sexual communication and self-efficacy between partners, including positive and risky topics on sexual communication, with scores ranging from 1 (*very difficult*) to 4 (*very easy*) (21). Participants were asked to choose the ease or difficulty of communicating about sex or sexual history, condom negotiation, positive and negative sexual messages, and contraceptive communication. No reverse-scored items were used. The higher the SCSES score, the more influential the participants' sexual communication, including sexual health, pleasure, and negotiation. The Cronbach's α in the original study was 0.93 (21); in the present study, it was 0.91, which showed high internal consistency. Permission to use the tool was obtained from the original author.

#### 2.3.2 Sexual assertiveness scale

Morokoff et al. (22) developed the original Sexual Assertiveness Scale (SAS) to measure sexual assertiveness, which includes factors such as initiation, refusal, pregnancy, and sexually transmitted disease (STD) prevention assertiveness with a regular partner. Lee and Lee (23) cross-culturally adapted the initial measurement, which is frequently used to measure Korean young adults' sexual self-assertiveness. In Lee and Lee's (23) study, the Korean version of the SAS comprised 21 items, scored on a 4-point Likert scale (1 = *strongly disagree*, 2 = *disagree*, 3 = *agree*, and 4 = *strongly agree*), with higher scores reflecting higher sexual self-assertiveness. Ten items (1, 3, 4, 6, 7, 8, 11, 12, 15, 17) were reverse scored. The Cronbach's α in the original study ranged from 0.66 to 0.86 (22), was 0.72 in the adapted study (23), and was 0.74 in the present study.

#### 2.3.3 Safe sex behavior questionnaire

The Safe Sex Behavior Questionnaire (SSBQ), initially developed by DiIorio et al. (24), comprises 24 items. Moon et al. (25) cross-culturally adapted this tool. This questionnaire includes questions on sexual behaviors, condom usage, high-risk sexual behaviors, and sexual communication and negotiation. It measures the frequency of safe sex practices by scoring them on a scale from 1 (*never*) to 4 (*always*). The reverse-scored items are 2, 7, 13, 14, 15, 20, 22, 23, and 24. A higher score indicates safer sexual behavior. The Cronbach's α ranged from 0.52 to 0.85 in the original study (the Cronbach's α of the complete scale was not provided) (24); it was 0.58–0.66 in Moon's study (the total scale's Cronbach's α was 0.73) (25), and the value was 0.74 in this study.

#### 2.3.4 Validation process

##### 2.3.4.1 Translation procedure

For cross-cultural adaptation, the SCSES was translated based on its usage in another country and language and its cultural aspects. The translation process followed the guidelines of Beaton et al. (26). Before the translation process, the authors explained the purpose of the study to the original authors and obtained their permission. The guidelines were divided into six stages. The first stage was forward translation. At this stage, two independent bilingual translators wrote the reports separately, which were translated into the target language. In the second stage, three experts, including the two translators from the first stage, discussed how to synthesize draft translations and made decisions regarding the draft translation by comparing their reports to resolve discrepancies. The third stage was the back-translation. Two translators fluent in English and Korean translated the questionnaire back into its original language. Here, the original and translated versions were compared and carefully reviewed to check for serious inconsistencies or conceptual errors in the translation; minor changes were made after discussion.

In the fourth stage, reports from previous stages were reviewed. A committee and a panel of three experts in reproductive health and women's health nursing also considered social and cultural equivalence from each stage. After discussions and agreements from all concerned parties, we produced a pre-final version of the KR-SCSES. Some words and phrases were modified to improve item equivalence. In the final stage, we pilot-tested the final version of the questionnaire. We recruited 10 college students and asked them to complete a questionnaire. After completion, the participants were asked to share their thoughts. Upon receiving feedback, minor changes were made to improve clarity, grammar, and readability. The final stage involved finalizing the measurements. Thus, we developed 20 items from the KR-SCSES.

#### 2.3.5 Content validity

The item-level content validity index (I-CVI) was used to test the content validity. Five experts graded the I-CVI using a 4-point Likert scale. As a result of the experts' evaluations, items with high relevance were evaluated as 3–4 points, and items with little or no relevance were assessed as 1–2 out of 1–4 points.

#### 2.3.6 Construct validity

##### 2.3.6.1 Exploratory factor analysis

Promax rotation was conducted below oblique rotation for the exploratory factor analysis (EFA). The Kaiser–Meyer–Olkin (KMO) measure and Bartlett's test of sphericity were used to assess sampling adequacy and evaluate factorability regarding the magnitude of intercorrelations. A screening test for eigenvalues was performed to determine the number of factors to be retained. Subsequently, the cumulative proportion (%) of variance was used to measure the percentage of variance accounted for by the current and preceding factors. Finally, the factor loading for each item on the factors was checked using a pattern matrix.

##### 2.3.6.2 Confirmatory factor analysis

A confirmatory factor analysis (CFA) was conducted to verify the factor structure that the EFA had defined and evaluate the validity of the measurement model. A factor loading >0.30 was considered reasonable, and all items were loaded into the factors in this study. We used the most explanatory and well-known indicators of model fit, namely, chi-square, *df* and its *p*-value, Steiger–Lind root mean square error of approximation (RMSEA) and its 90% confidence interval (CI), minimum discrepancy and discrepancy divided by degree of freedom (CMIN/DF), root mean square residual (RMR), Tucker–Lewis index (TLI), and Bentler comparative fit index (CFI).

##### 2.3.6.3 Criterion-related validity

Criterion-related validity was measured by comparing the Korean SAS and SSBQ with the KR-SCSES, widely used to measure sexual behavior in young adults. By calculating the correlation between the results of these measurements, we concluded that these measures predict a concrete outcome, implying that our test will operate correctly if there is a high correlation.

#### 2.3.7 Internal consistency reliability

Internal consistency was estimated by calculating the inter-item correlation, item-total correlation (more than 0.4 indicating good internal consistency), Cronbach's α coefficients (more than 0.6 indicating acceptable internal consistency), and Cronbach's α if the item was deleted.

#### 2.3.8 Data analyses

Statistical analyses were conducted using SPSS Windows software version 25.0 and AMOS Win 25.0 program.

## 3 Results

### 3.1 General characteristics of participants

Table 1 presents the participants' general characteristics. The findings showed that the sample comprised approximately equal proportions of males and females (50.7 and 49.3%, respectively). The sexual orientation of the participants was primarily heterosexual (*n* = 193, 85%). Their average age was 22.77 [standard deviation (*SD*) = 2.420] years, ranging from 18 to 29 years. More than 90% of the participants were enrolled in a bachelor's degree course. Approximately 4 out of 10 were seniors (*n* = 97, 42.7%), followed by juniors (*n* = 53, 23.3%) and sophomores (*n* = 51, 22.5%), of which more than half were majoring in science and technology and humanities (41 and 22%, respectively). Most participants were not religious (*n* = 166, 73.1%).

**Table 1:** General characteristics of participants (*N* = 227).

**Characteristic**	**Category**	***n***	**(%)**	**Mean ±*SD* (min–max)**
Sex	Female	115	50.7	
	Male	112	49.3	
Sexual orientation	Bisexual	30	13.2	
	Homosexual (lesbian, gay)	4	1.8	
	Heterosexual	193	85.0	
Age (years)				22.77 ± 2.420 (18–29)
College year	Associate degree course	21	9.3	
	Bachelor's degree course	206	90.7	
Grade	Freshman	26	11.5	
	Sophomore	51	22.5	
	Junior	53	23.3	
	Senior	97	42.7	
Major	Humanities	50	22.0	
	Social science	33	14.5	
	Science and technology	93	41.0	
	Health and medical (including nursing)	22	9.7	
	Arts and physical education	25	11.0	
	Other (human ecology and education)	4	1.8	
Religion	None	166	73.1	
	Catholic	12	5.3	
	Christian	38	16.7	
	Buddhism	11	4.8	
Age at first sexual intercourse (years)	≤ 17	16	7.0	20.11 ± 2.168 (17–29)
	18–20	140	61.7	
	21–24	59	26.0	
	≥25	12	5.3	
Frequency of contraceptive use in 6 months	Rarely	17	7.5	
	Sometimes	18	7.9	
	Often	38	16.7	
	Always	154	67.8	
Primary decision-maker to use contraceptive for 6 months	Myself	64	28.2	
	Partner	5	2.2	
	Mutual agreement	158	69.6	
Number of sexual partners in 6 months	One	181	79.7	
	Two	27	11.9	
	Three	11	4.8	
	More than four	8	3.6	
Types of sexual partners in 6 months^*^	Reliable (consistent) partner	212	88.3	
	Casual partner	19	7.9	
	Unexpected (one-time, one-night) partner	9	3.8	
Have had STDs	Yes	11	4.8	
	No	216	95.2	
You or your partner have had unwanted pregnancy	Yes	4	1.8	
	No	223	98.2	
Sexual tolerance	Conservative	21	9.3	
	Neutral	132	58.1	
	Open	74	32.6	
Sexual subjectivity	Passive	28	12.3	
	Neutral	133	58.6	
	Proactive	66	29.1	
Experience of unwanted sex	Yes	16	7.0	
	No	211	93.0	
Ability to communicate and negotiate with partners in sexual behavior or relationship	Low	5	2.2	
	Middle	108	47.6	
	High	114	50.2	
Sexual communication satisfaction with partner	Not satisfied	5	2.2	
	Neutral	37	16.3	
	Satisfied	136	59.9	
	Vary satisfied	49	21.6	
Communication type	Open (democratic): knowing each other well and interacting effectively in communication	187	82.4	
	Blindly (assertive): asserting one's point in communication and ignoring the opinions of others	9	4.0	
	Potential (reticent): not revealing one's feelings and attitudes in communication and accepting the other's opinions partially	31	13.7	
Sexual satisfaction during a sexual behavior or relationship^a^	Bad to good			5.61 ± 1.182 (1–7)
	Unpleasant to pleasant			5.61 ± 1.215 (1–7)
	Negative to positive			5.65 ± 1.166 (1–7)
	Unsatisfying to satisfying			5.53 ± 1.224 (1–7)
	Worthless to valuable			5.59 ± 1.260 (1–7)
Frequency of you and your partner discussing about below topics^b^	How to prevent pregnancy			3.60 ± 0.754 (1–4)
	How to use condoms			3.52 ± 0.848 (1–4)
	How to prevent STDs or AIDS virus			2.91 ± 1.079 (1–4)
	Partner's sex history			2.79 ± 1.121 (1–4)

^*^Multiple choice.

Sexual tolerance: the degree of tolerance for kissing, caressing, and sexual intercourse according to the level of physical contact; sexual subjectivity: one's will or attitude in sexual behavior or relationship; sex history: have had sexual experiences, engaged in certain sexual activities, and so on.

^a^Global Measure of Sexual Satisfaction of Korean Version.

^b^Korean Partner Communication Scale.

The questionnaires also asked about their sexual behavior and relationships. The average age of the first sexual intercourse was 20.11 (*SD* = 2.168) years, and most participants were between 18 and 20 years at the time of their first sexual intercourse. The results showed that 68% had always used contraceptives in the last 6 months, and the primary decision-makers were themselves and their partners (*n* = 158, 69.6%). The participants mostly had one sexual partner in the past 6 months (*n* = 181, 79.7%), and their sexual partners were consistent (*n* = 212, 88.3%). More than 90% of the participants did not experience STDs, unwanted pregnancies, or unwanted sex. They answered that their sexual tolerance and subjectivity were neutral (*n* = 132, 58.1% and *n* = 133, 58.6%, respectively), followed by open (*n* = 74, 32.6%) and proactive (*n* = 66, 29.1%). More than half of the participants had a high ability to communicate and negotiate with sexual partners (*n* = 114, 50.2%) and had open discussions about sexual behavior and relationships (*n* = 187, 82.4%).

The average mean score of the Korean Version of the Global Measure of Sexual Satisfaction was obtained by summing each item using a 7-point Likert-type scale: good–bad (5.61 points, *SD* = 1.182), pleasant–unpleasant (5.61 points, *SD* = 1.215), positive–negative (5.65 points, *SD* = 1.166), satisfying–unsatisfying (5.53 points, *SD* = 1.224), and valuable–worthless (5.59 points, *SD* = 1.260), with higher scores indicating greater sexual satisfaction during a sexual relationship with their partner for each subcategory. The topics of preventing pregnancy (3.60 points, *SD* = 0.754) and how to use condoms (3.52 points, *SD* = 0.848) were frequently discussed with partners.

### 3.2 Validity of the Korean version of the sexual communication self-efficacy scale

#### 3.2.1 Content validity

In this study, two items were evaluated as having 1 or 2 points: Items 6 (demand that a condom be used) and 16 (discuss how to put on a condom). For an item with a score of < 1, the question was corrected or deleted through discussions. We decided to delete these two items after an in-depth discussion and consensus. These items were omitted because of the influence of Korean grammar as their meaning overlapped with other questions in the direct translation from English to Korean. Subsequently, the I-CVI was reevaluated, and the average value of the I-CVI was 0.85. Thus, 18 items were selected to test validity and reliability.

### 3.2.2 Construct validity test

#### 3.2.2.1 Part 1: item analysis for internal consistency

Table 2 shows the item-to-total correlations of the KR-SCSES. The findings showed that this tool was considered acceptable. No items were lower than the standard, which was < 0.30 in the item-total correlation. There was no significant effect on internal consistency even when the item was removed for all items. Therefore, all items were used to test psychometric properties without deleting any.

**Table 2:** Internal consistency (corrected item-total correlation) and mean (standard deviation) (*N* = 227).

**Item**	**Content (Do you find it difficult to…)**	**Item-total correlation**	**Alpha if item deleted**	**Mean ±*SD***
KR-SCSES 1	Ask how many partners they have had?	0.511	0.907	2.42 ± 0.915
KR-SCSES 2	Ask if they have ever shared needles?	0.470	0.909	2.88 ± 0.928
KR-SCSES 3	Ask if they are having sex with other people?	0.531	0.907	2.63 ± 1.020
KR-SCSES 4	Aski if they have ever had a sexually transmitted infection?	0.540	0.906	2.48 ± 0.961
KR-SCSES 5	Ask if a condom could be used for sex with them?	0.507	0.907	3.34 ± 0.768
KR-SCSES 6	Refuse to have sex if they won't use a condom?	0.513	0.907	3.15 ± 0.780
KR-SCSES 7	Tell them a certain sexual activity hurts you?	0.575	0.905	3.21 ± 0.745
KR-SCSES 8	Tell them if a certain sexual activity makes you uncomfortable?	0.615	0.904	3.09 ± 0.762
KR-SCSES 9	Tell them that a certain sexual activity is not making you feel good?	0.639	0.903	3.00 ± 0.793
KR-SCSES 10	Tell them you do not want to have sex?	0.501	0.907	3.05 ± 0.708
KR-SCSES 11	Suggest a new sexual activity (e.g., a new sexual position)?	0.553	0.906	2.83 ± 0.799
KR-SCSES 12	Tell them you would like to have sex more often?	0.622	0.904	2.92 ± 0.789
KR-SCSES 13	Tell them that a sexual activity feels good?	0.582	0.905	3.15 ± 0.775
KR-SCSES 14	Tell them that you want to have sex?	0.652	0.903	2.99 ± 0.812
KR-SCSES 15	Tell them you like a specific sexual activity?	0.647	0.903	2.90 ± 0.817
KR-SCSES 16	Initiate sex?	0.645	0.903	2.93 ± 0.872
KR-SCSES 17	Talk about how it feels to use a condom?	0.628	0.904	3.04 ± 0.813
KR-SCSES 18	Talk about whether a condom is on correctly?	0.639	0.904	3.19 ± 0.747

#### 3.2.2.2 Part 2: exploratory factor analysis

Table 3 presents the results of the EFA. The Promax rotation of the KR-SCSES items generated three factors that explained the results and accounted for 62.26% of the variance. No item was deleted because all items were loaded onto one factor, and the loading weight was more than 0.30. The loading ranges were 0.41–0.87, 0.68–0.89, and 0.52–0.88 for Factors 1–3, respectively.

**Table 3:** Results of exploratory factor analysis (patterned matrix).

**Item**	**Factor**		
	**1**	**2**	**3**
KR-SCSES 8	0.868		
KR-SCSES 9	0.773		
KR-SCSES 6	0.742		
KR-SCSES 5	0.739		
KR-SCSES 7	0.723		
KR-SCSES 18	0.558		
KR-SCSES 17	0.473		
KR-SCSES 10	0.412		
KR-SCSES 14		0.878	
KR-SCSES 15		0.842	
KR-SCSES 12		0.800	
KR-SCSES 16		0.733	
KR-SCSES 11		0.689	
KR-SCSES 13		0.679	
KR-SCSES 3			0.884
KR-SCSES 1			0.754
KR-SCSES 4			0.737
KR-SCSES 2			0.523
Eigenvalue	7.306	2.148	1.754
Total variance explained proportion (%)	40.587	11.931	9.744
Cumulative proportion (%)	40.587	52.518	62.262

Bartlett's sphericity test was significant (χ^2^ = 2,174.475, *df* = 153, *p* < 0.001), and the KMO measure was 0.91, indicating a good fit for the factor analysis. Factors with eigenvalues ≥1 were extracted, and the slope of the scree plot significantly decreased after the component with the value of 2.

The first factor contained eight items (5, 6, 7, 8, 9, 10, 17, and 18), which could be interpreted as a dimension of “reluctance and unwillingness” (eigenvalue, 7.31; variance explained, 40.59%). The second factor consisted of six items (11–16) named “suggestion and recommending” (eigenvalue, 1.89; variance explained, 11.93%). Finally, the third factor was labeled “personal history” and comprised four items (1–4) (eigenvalue, 1.75; variance explained, 9.74%).

#### 3.2.2.3 Part 3: confirmatory factor analysis

Table 4 presents the results of the CFA. The model fit of KR-SCSES was χ^2^ = 283.444 (*df* = 132, *p* < 0.001), RMSEA = 0.071 (90% CI: 0.060–0.083), CMIN/DF = 2.147, RMR = 0.043, TLI = 0.916, and CFI = 0.928.

**Table 4:** Model's goodness of fit (appropriateness of confirmatory factor analysis).

**Model**	**χ^2^**	***df***	**Chi-square *p*-value**	**RMSEA (90% CI)**	**CMIN/DF**	**RMR**	**TLI**	**CFI**
	283.444	132	< 0.001	0.071 (0.060–0.083)	2.147	0.043	0.916	0.928

Overall, the results indicated that the all-inclusive model was a good-fit model that adequately described our model by meeting all the recommended criteria, excluding the chi-square *p*-value (if the chi-square test is not significant).

**Figure 1:**
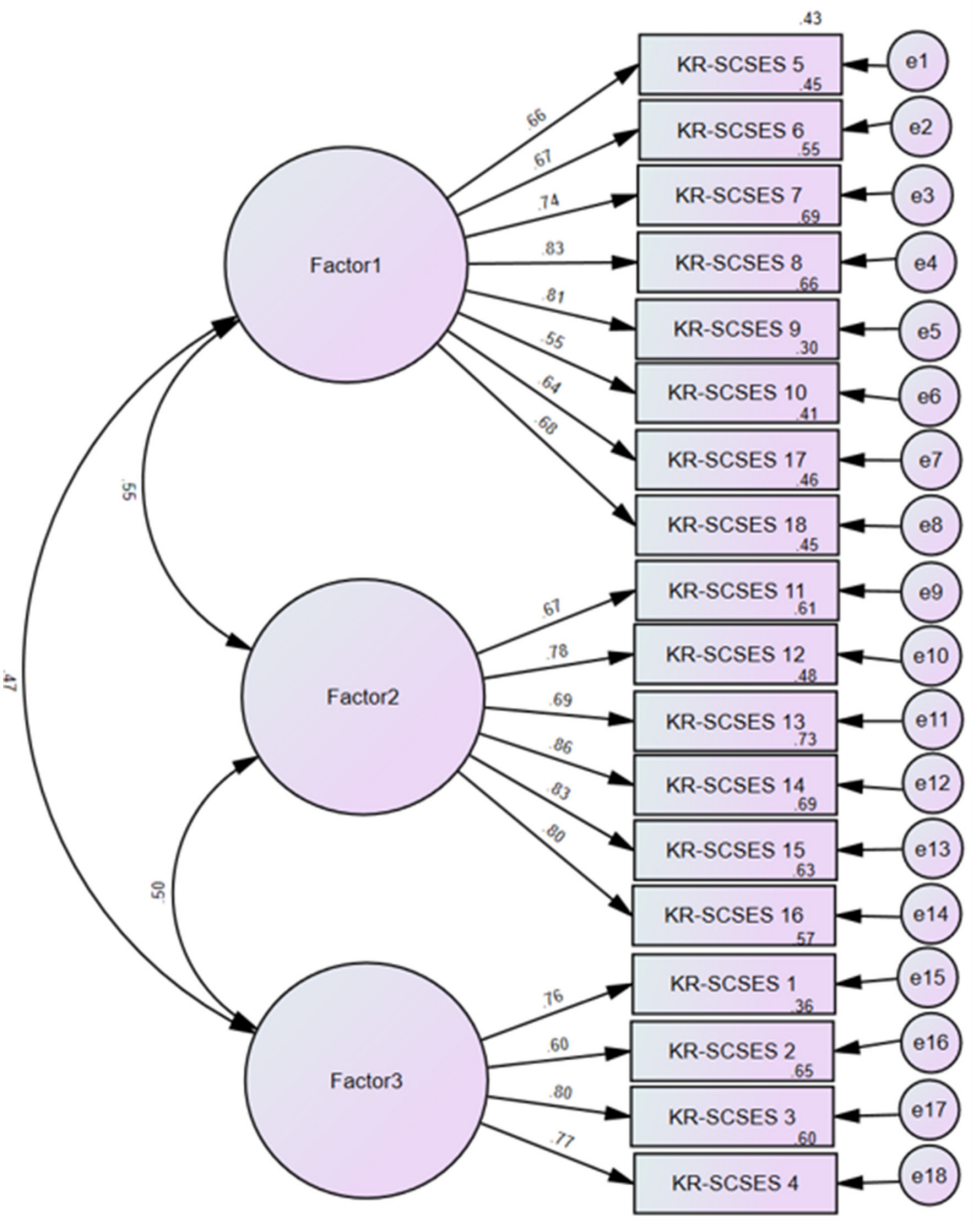
Path diagram with standardized estimates.

Furthermore, considering the path diagram for the CFA model shown in Figure 1, all items were highly loaded on each factor and were above 0.4. The factor loadings ranged from 0.55 to 86. The highest factor-loading items for each factor were KR-SCSES 8 (0.81) in Factor 1, KR-SCSES 14 (0.84) in Factor 2, and KR-SCSES 3 (0.80) in Factor 3. The lowest factor-loading items were KR-SCSES 10 (0.55), KR-SCSES 11 (0.67), and KR-SCSES 2 (0.60).

#### 3.2.2.4 Part 4: criterion validity

The relationships between the KR-SCSES, SAS-K, and SSBQ-K were examined. All three instruments were positively correlated and statistically significant (KR-SCSES and SAS-K *r* = 0.495; KR-SCSES and SSBQ-K *r* = 0.475; and SAS-K and SSBQ-K *r* = 0.536, *p* for all < 0.001).

### 3.2.3 Reliability of the Korean version of the sexual communication self-efficacy scale

The Cronbach's α values were 0.91 for the 18 items on the KR-SCSES (Table 5). For “reluctance and unwillingness” (Factor 1), the Cronbach's α was 0.88; for “suggestion and recommending” (Factor 2), the Cronbach's α was 0.90; and for “personal history” (Factor 3), the Cronbach's α was 0.82. These results indicated high reliability (27).

**Table 5:** Criterion validity and reliability.

	**(1)**	**(2)**	**(3)**	**Cronbach's alpha**	
KR-SCSES (1)	–			0.91	Factor 1 0.88 Factor 2 0.90 Factor 3 0.82
SAS-K (2)	0.495 (< 0.001)	–		0.74	
SSBQ-K (3)	0.475 (< 0.001)	0.536 (< 0.001)	–	0.74	

## 4 Discussion

College students in situations involving sexual behavior require appropriate assessment and intervention programs and policies to enable them to engage in their desired sexual behavior, reject unwanted sexual behavior, avoid getting pregnant, and prevent diseases. This necessitates the development of an evaluation tool for accurately measuring communication and self-efficacy, which are important variables of sexual behavior that can be comprehensively measured. However, in South Korea, no tool can directly measure these variables, and most studies have used tools developed abroad. Using a measurement tool designed for various participants from different social and cultural backgrounds without verification of reliability and validity has limitations in the systematic analysis of the study results, thereby requiring a detailed analysis.

To determine the appropriateness of applying the SCSES tool to Korean college students, the reliability and validity of the KR-SCSES were verified in a previous study by Quinn-Nilas et al. (21). We judged the validity and reliability of the KR-SCSES were judged by evaluating the content validity, construct validity, criterion validity, and reliability coefficient of the cross-culturally adapted SCSES. Content validity was established by performing facial validity tests during the translation process and using the I-CVI of subject matter experts. Construct validity was evaluated using the EFA and CFA. Subsequently, criterion validity was assessed using the SAS-K and SSBQ-K, which measured participants' sexual assertiveness and sexual behavior. The overall reliability of the KR-SCSES was evaluated using the Cronbach's α for the sub- and total scales.

Content validity is vital for determining whether the item properly represents the domain measured by the instrument. A total of 10 Korean college students evaluated the face effectiveness of the KR-SCSES. Facial validity may not be accurate; therefore, exploring how potential participants understand the tool is crucial. We made minor changes by reflecting on the participants' opinions. This process improves the readability of the instrument; therefore, it is judged to be highly applicable in future research targeting college students.

Furthermore, subject matter experts are essential for determining the relevance and appropriateness of the tool's items. Here, the I-CVI quantifies the degree of consensus among five subject matter experts. If the I-CVI score is ≤ 0.5, it is judged that there is no content validity, and if it is more than or equal to 0.80, the content validity is considered to be high. In the process of direct translation (from English to Korean), the meaning of the two items overlapped with that of the other questions; therefore, two questions were deleted. The decision was made after two in-depth discussions and consultations among the researchers and subject matter experts who participated in the I-CVI evaluation. The average I-CVI was as high as 0.85, confirming that the KR-SCSES was composed of valid content to measure sexual communication and self-efficacy.

Construct validity evaluates how the items and areas match the theoretical constructs defined in the KR-SCSES. The EFA and CFA assessed the construct validity of the KR-SCSES. The original tool is relatively recent and was developed in the United States. Both EFA and CFA were performed to ensure that the tool was suitable for application to Korean university students. The EFA is a data-driven statistical tool for determining the association of items and components in a tool and enables hypothesis generation in the early stages of tool development. By contrast, the CFA is a theory-driven complex statistical approach to establish construct validity.

In this study, the EFA derived a 3-factor solution with 18 items. The EFA's simple-structure guidelines were followed when determining the three KR-SCSES factors. Each factor generated at least three items with a coefficient load < 0.30, which met the assumption of EFA use. The KMO and Bartlett's sphericity tests provided information about the item's factor by checking the item correlation and item sampling adequacy, respectively. Items with low correlation indicated non-facticity. The item-to-total correlation and KMO and Bartlett tests showed sufficient item facticity in the KR-SCSES.

These results showed that the sample was suitable for factor analysis. The sample size was 227, ~12 times or more than the number of items (20 items), and a sufficient sample size of 200 or more was used. The criterion of eigenvalue 1.0 or higher, which is most commonly applied to determine the appropriate number of factors in factor extraction, was also satisfied. The extracted factors generally met the criterion to explain at least 60% of the total variance.

We also used various model fit indices, such as chi-square, RMSEA, CMIN/DF, RMR, TLI, and CFI, to determine whether the CFA provides evidence for the tool's theoretical fit. The χ^2^ value was used to check whether the model and data matched in the CFA. It is desirable when the *p*-value is >0.05; however, when the sample size becomes large, it is < 0.05 in most cases. The RMSEA values ranging from 0.05 to 0.08 are acceptable, and CMIN/DF values ≤ 3 are fair. No absolute criterion exists for the acceptance level of RMR, but the closer it is to 0, the better the fit. Moreover, TLI and CFI should be at least 0.70; if above 0.90, the values indicate that the model's fit is optimal.

This study's results were suitable for all criteria except for the *p*-value of the chi-square test; therefore, the model was judged to be relatively suitable. The original tool was developed in 2015, and no further research has been conducted to verify its validity and reliability by adapting it worldwide. As no study has reported validity verification, it is meaningful because construct validity was confirmed by running the EFA and CFA. The tool's validity must be verified by expanding the research subject and measuring it repeatedly for its efficient application.

The SAS-K and SSBQ-K were used as references for criterion validity verification; they showed a statistically significant correlation of 0.50 and 0.48 between college students' sexual communication and self-efficacy measurement tools, respectively. However, the concepts used in those measurements cannot be regarded as the same as those in this study. As mentioned before, some tools separate and measure sexual behavior, sexual communication, and sexual self-efficacy, but no tool currently measures each comprehensively. Thus, this study's criterion validity was verified through each tool. Therefore, the lack of an accurate criterion validity analysis was considered a limitation of this study.

The reliability of the KR-SCSES was established by determining its Cronbach's α. This approach is suitable for establishing the reliability of all instruments because it evaluates the multidimensional nature of the tool. Regarding reliability verification, a reliability coefficient of 0.90 is generally interpreted as “excellent”, 0.80 as “good”, 0.70 as “reasonable”, and 0.50 or less as not recommended to be used. Ultimately, the reliability of each factor was confirmed. In this study, the Cronbach's α value of the KR-SCSES was 0.82–0.90 for each subcategory, and the average reliability value was high at 0.91. Therefore, it can be evaluated as a measurement tool with a high internal consistency for measuring sexual communication and self-efficacy. This tool's reliability should be confirmed in further studies. Although this tool was abbreviated to 3 factors and 18 questions, its internal consistency was maintained; therefore, it was judged to be a reliable tool for measuring the sexual communication and self-efficacy of Korean college students.

The KR-SCSES was reduced to 18 items through the EFA and CFA; however, the internal consistency of the tool was maintained. Therefore, the KR-SCSES was verified as a reliable and valid measurement tool for measuring the sexual behavior of college students to attempt their desired sexual behavior, reject unwanted sexual behavior, avoid getting pregnant, and prevent disease. The reduction in items has improved the readability and ease of measurement, which busy college students can easily measure. In the future, repeated measurement and verification can be conducted among university students to increase the reliability and validity of the diagnosis of this scale. Additionally, this tool can be evaluated in nursing policy and educational research.

This study has several limitations. First, the KR-SCSES was developed and validated exclusively with 227 South Korean college students, limiting its generalizability to other populations, including non-college-attending youth and individuals from diverse cultural or socioeconomic backgrounds. Second, the scale measures self-efficacy but not actual communication behaviors, which may not always align with the reported confidence. Finally, the reliance on self-reported data introduces the possibility of social desirability bias, with no verification of whether the self-efficacy scores reflect actual behaviors. Future research should address these limitations by testing the scale with broader populations and incorporating more objective measures.

## 5 Conclusions

In this study, we adapted using a thorough translation of the tool developed by Quinn-Nilas et al. (21) to construct the KR-SCSES and verify the reliability and validity of the tool. We intended to prepare the grounds for the tool's domestic applicability and expand its use by targeting college students. Furthermore, after verifying its construct validity and reliability using the EFA and CFA, the SCSES measurement tool adopted in Korea comprised 3 factors and 18 items. By factor, there were eight items for reluctance and unwillingness, six for suggestion and recommendation, and four for personal history. Thus, the tool's validity was secured as all items appeared to explain each factor included in the item well. As a result of checking the internal consistency and stability of the tool, reliability was secured. The construct validity and internal consistency were verified, confirming the applicability of the KR-SCSES as a tool for measuring sexual communication skills and self-efficacy in the sexual behavior of university students in Korea. Therefore, the KR-SCSES is expected to revitalize related research by considering efficient educational and institutional measures to inhibit dangerous sexual behaviors and prevent sexual problems among domestic college students.

## Data Availability

The original contributions presented in the study are included in the article/supplementary material, further inquiries can be directed to the corresponding author.

## References

[1] Kim HY. Factors affecting contraceptive attitude of college students. J Korea Acad Indust Cooper Soci. (2019) 20:384–93. 10.5762/KAIS.2019.20.5.384

[2] Cornelius T Kershaw T. Perception of partner sexual history: effects on safe-sex intentions. Health Psychol. (2017) 36:704–12. 10.1037/hea0000474 28318276 PMC5476493

[3] Victor EC Hariri AR. A neuroscience perspective on sexual risk behavior in adolescence and emerging adulthood. Dev Psychopathol. (2016) 28:471–87. 10.1017/S0954579415001042 26611719 PMC4828296

[4] Fehr SK Vidourek RA King KA Nabors LA. Relationship factors' impact on condom use among college students. Sex Cult. (2018) 22:724–39. 10.1007/s12119-018-9503-9

[5] Mehra D Östergren PO Ekman B Agardh A. Inconsistent condom use among Ugandan university students from a gender perspective: a cross-sectional study. Glob Health Action. (2014) 7:1–9. 10.3402/gha.v7.22942 24725363 PMC3984407

[6] Caldwell K Mathews A. The role of relationship type, risk perception, and condom use in middle socioeconomic status black women's HIV-prevention strategies. JBSR. (2015) 2:91–120. 10.1353/bsr.2016.0002 29218311 PMC5716635

[7] Eleftheriou A Bullock S Graham CA Stone N Ingham R. Does attractiveness influence condom use intentions in heterosexual men? An experimental study. BMJ Open. (2016) 6:e010883. 10.1136/bmjopen-2015-010883 27315834 PMC4916619

[8] Lee JY. Factors affecting contraceptive use among adolescent girls in South Korea. Child Health Nurs Res. (2017) 23:259–67. 10.4094/chnr.2017.23.3.259

[9] Lee EM Kim KY. The effect of sexual attitude, sexual attitude of parents, and depression on sexual intercourse experience of university students. Korean J Stress Res. (2017) 25:155–61. 10.17547/kjsr.2017.25.3.155

[10] Matera C. Encouraging safer sex: mediating and moderating effects on condom use among Italian girls. Int J Sex Health. (2014) 26:217–28. 10.1080/19317611.2013.858803

[11] Bryan AEB Norris J Abdallah DA Zawacki T Morrison DM George WH. Condom-insistence conflict in women's alcohol-involved sexual encounters with a new male partner. Psychol Women Q. (2017) 41:100–13. 10.1177/0361684316668301 29720782 PMC5927388

[12] Motsomi K Makanjee C Basera T Nyasulu P. Factors affecting effective communication about sexual and reproductive health issues between parents and adolescents in Zandspruit informal settlement, Johannesburg, South Africa. Pan Afr Med J. (2016) 25:1–7. 10.11604/pamj.2016.25.120.9208 28292083 PMC5325495

[13] Somersa CL Anagurthia C. Parents' attitudes about adolescents' premarital sexual activity: the role of inter-parent consistency/inconsistency in sexual outcomes. Health Educ J. (2013) 73:545–53. 10.1177/0017896913506702

[14] Hwang SW Chung C. Structural equation modeling on contraception behavior of unmarried men and women in Korea: Gender difference. J Korean Acad Nurs. (2014) 44:159–69. 10.4040/jkan.2014.44.2.159 24859121

[15] Kim KH Cho EN. Reliability and validity of the Korean version of the contraceptive self-efficacy scale: Focused on women university students. Korean J Women Health Nurs. (2016) 22:151–61. 10.4069/kjwhn.2016.22.3.151 37684864

[16] Brasileiro J Widman L Evans R Javidi H. Social self-efficacy and sexual communication among adolescents in the United States: a cross-sectional study. Sex Health. (2021) 18:172–9. 10.1071/SH20221 33926613 PMC11926737

[17] Ferrer-Urbina R Sepúlveda-Páez GL Henríquez DT Acevedo-Castillo DI Llewellyn-Alvarado DA. Development and validity evidence of the multidimensional scale of sexual self-concept in a Spanish-speaking context. Psicol-Reflex Crí*t*. (2019) 32:1–8. 10.1186/s41155-019-0136-1 32027012 PMC6966990

[18] Baele J Dusseldorp E Maes S. Condom use self-efficacy effect on intended and actual condom use in adolescents. J Adoles Health. (2017) 28:421–31. 10.1016/S1054-139X(00)00215-9 11336873

[19] Sayyadi F Golmakani N Ebrahimi M Saki A Karimabadi A Ghorbani F. Determination of the effect of sexual assertiveness training on sexual health in married women: a randomized clinical trial. Iran J Nurs Midwifery Res. (2019) 24:274–80. 10.4103/ijnmr.IJNMR_51_17 31333741 PMC6621498

[20] DeVellis RF. Scale Development: Theory and Applications. Los Angeles, CA: Sage (2017).

[21] Quinn-Nilas C Milhausen RR Breuer R Bailey J Pavlou M DiClemente RJ. et al. Validation of the sexual communication self-efficacy scale. Health Educ Beh. (2016) 43:165–71. 10.1177/1090198115598986 26286296

[22] Morokoff PJ Quina K Harlow LL Whitmire L Grimley DM Gibson PR. Sexual assertiveness scale for women: development and validation. J Pers Soc Psychol. (1997) 73:790–804. 10.1037/0022-3514.73.4.790 9325594

[23] Lee JY Lee ES. Development and validation of the Korea sexual self-assertiveness scale for female. Korean J Counsel. (2006) 7:47–62.

[24] DiIorio C Parsons M Lehr S Adame D Carlone J. Measurement of safe sex behavior in adolescents and young adults. Nurs Res. (1992) 41:203–8. 10.1097/00006199-199207000-00003 1408860

[25] Moon SJ. Sexual health knowledge, perceived social support, and safe sex behavior among international students in Korea (Master's thesis). Yonsei University, Seoul, South Korea (2017).

[26] Beaton DE Bombardier C Guillemin C Ferraz MB. Guidelines for the process of cross-cultural adaptation of self-report measures. Spine. (2000) 25:3186–91. 10.1097/00007632-200012150-00014 11124735

[27] Hinton PR Brownlow C McMurray I Cozens B SPSS. Explained. London: Routledge (2004).

